# Desloratadine Induces *TP53*-Dependent Apoptosis in MCF-7 Breast Cancer Cells

**DOI:** 10.3390/cells14211725

**Published:** 2025-11-03

**Authors:** Syed Rashel Kabir, Taufique Abdullah, Gausul Azam, Tamzid Hossain Molla, Hasan Ali, Mojnu Miah, Mohammad Taufiq Alam, Sayem Miah

**Affiliations:** 1 Department of Biochemistry and Molecular Biology, University of Rajshahi, Rajshahi 6025, Bangladesh; taufiqueabdullah632@gmail.com (T.A.); gausulazam.bmb@gmail.com (G.A.); mdhasanaliru2@gmail.com (H.A.); 2 Department of Applied Chemistry and Chemical Engineering, University of Rajshahi, Rajshahi 6205, Bangladesh; thmolla@ru.ac.bd (T.H.M.); talam@ru.ac.bd (M.T.A.); 3 Department of Biochemistry and Molecular Biology, College of Medicine, Winthrop P. Rockefeller Cancer Institute (WPRCI), University of Arkansas for Medical Sciences (UAMS), Little Rock, AR 72205, USA; mmiah@uams.edu

**Keywords:** drug repurpose, breast cancer, desloratadine, TP53 gene, apoptosis

## Abstract

Breast cancer remains a leading cause of mortality among women despite advances in early detection and targeted therapies, underscoring the need for safer and more effective treatment options. Drug repurposing offers a promising strategy by leveraging existing pharmacological agents with established safety profiles. Desloratadine, a second-generation H1-histamine receptor antagonist widely prescribed for allergic conditions, has attracted interest in oncology because histamine signaling influences proliferation, angiogenesis, and immune responses, yet its anticancer potential remains poorly understood. In this study, we investigated its effects in MCF-7 breast cancer cells, which harbor wild-type *TP53*. Desloratadine inhibited cell viability in a dose-dependent manner, with an IC_50_ of 14.2 µg/mL. Mechanistic analyses revealed that growth inhibition was primarily mediated through apoptosis, confirmed by Hoechst 33342 staining, ROS generation, annexin V/PI staining, and caspase-dependent pathways. Gene expression profiling demonstrated upregulation of *TP53*, *FAS*, and *BAX*, alongside reduced *PARP-1* and *NF-κB* expression, with no detectable *STAT3* or *BCL2* expression. Flow cytometry indicated accumulation of cells in the sub-G_1_ phase and G_2_/M arrest, consistent with apoptosis induction. Molecular docking further supported these findings, showing that Desloratadine binds with high affinity to p53 (−7.0 kcal/mol), FAS (−6.8 kcal/mol), and NF-κB (−6.5 kcal/mol), forming stabilizing hydrogen bonds and hydrophobic interactions aligned with the observed gene expression changes. To confirm the functional role of *TP53*, we generated CRISPR-Cas9 knockout MCF-7 cells. Compared with wild-type cells, these knockout cells displayed markedly reduced sensitivity to Desloratadine, with the IC_50_ shifting from 14.2 µg/mL to 36.4 µg/mL, demonstrating that p53 is a key mediator of the drug’s cytotoxic effect. Collectively, these findings identify Desloratadine as a potential repurposed drug candidate for breast cancer therapy, acting at least in part through a p53-dependent apoptotic pathway.

## 1. Introduction

Breast cancer is one of the most common cancers worldwide and remains a leading cause of death [1]. Although early detection and targeted therapies have improved patient outcomes, conventional treatments are still limited by resistance, toxicity, and lack of long-term efficacy [2,3]. These limitations highlight the need for safer and more effective alternatives. One approach gaining increasing attention is drug repurposing, which explores new therapeutic applications for approved drugs. Repurposed agents have the advantage of established safety and pharmacological profiles, shorter development timelines, and lower costs compared with conventional drug discovery [4,5].

Several non-oncologic drugs have already demonstrated promising anticancer effects. For example, metformin, an antidiabetic agent, has been shown to inhibit tumor growth and improve treatment outcomes in various cancers through modulation of cellular metabolism and AMPK activation [6,7]. Niclosamide, an antihelminthic drug, has been reported to suppress key oncogenic pathways such as Wnt/β-catenin, STAT3, and NF-κB signaling [8,9]. Similarly, bicalutamide, an antiandrogen, is being used off-label in certain subtypes of breast cancer characterized by androgen receptor expression [10,11]. Because of this success, scientists are also studying other common, non-cancer drugs, like the allergy medicine Desloratadine, to see if they can also fight cancer.

Desloratadine (C19H19ClN2), a second-generation H1-histamine receptor antagonist, is widely prescribed as an anti-allergic and anti-inflammatory drug [12,13]. Beyond its well-known anti-allergic role, recent studies suggest that histamine signaling contributes to cancer progression through the regulation of proliferation, angiogenesis, and immune responses [14]. Activation of the histamine H_1_-receptor (HRH1) drives oncogenic progression by engaging key survival cascades, including the ERK and NF-κB signaling pathways, which collectively enhance tumor cell growth and confer resistance to programmed cell death [15,16]. Consequently, blockade of the H1 receptor by Desloratadine could suppress these pro-tumorigenic signals, thereby exerting direct anticancer effects and providing a clear rationale for investigation.

Moreover, breast cancer cells are particularly relevant targets because they are highly responsive to histamine signaling. Histamine receptors are overexpressed in both ER-positive and triple-negative subtypes, which correlates with enhanced proliferation, migration, and inflammatory cytokine production [14,17,18,19]. This makes breast cancer a biologically relevant model to investigate antihistamines like Desloratadine. Crucially, Desloratadine’s action aligns with key cancer drivers: it modulates chronic inflammation and oxidative stress through the inhibition of NF-κB and cytokine release that are strongly linked to breast cancer initiation and progression [20]. Therefore, Desloratadine holds the potential to interfere with multiple, convergent cancer-promoting pathways specific to breast cancer biology.

From a pharmacokinetic perspective, Desloratadine displays high oral bioavailability (~75–80%), extensive tissue distribution, and a plasma half-life of approximately 27 h, supporting sustained receptor engagement [21]. Clinically, Desloratadine is well-tolerated even at doses several-fold higher than standard therapeutic levels, with minimal cardiotoxic or hepatotoxic effects reported [22]. These safety and pharmacokinetic characteristics make Desloratadine an attractive candidate for repurposing, offering the possibility of achieving biologically relevant concentrations in tumor tissues without significant systemic toxicity. However, its direct anticancer efficacy and molecular mechanisms, particularly in breast cancer cells, remain largely unexplored.

Within the network of key cellular regulators, the tumor suppressor p53 plays a pivotal role by controlling cell cycle arrest, DNA repair, and apoptosis under cellular stress conditions. Changes in p53 are found in about half of all human cancers, showing how important it is in cancer development [23]. The MCF-7 breast cancer cell line is estrogen receptor–positive and has wild-type p53, which makes it a good model to study p53-dependent effects [24]. If Desloratadine’s anticancer effect depends on p53, it can give important clues about how it works and its possible use as therapy.

In the present study, we investigated the anticancer activity of Desloratadine in the MCF-7 breast cancer cell line. Multiple approaches were employed, including MTT viability assays, flow cytometry, cell cycle analysis, fluorescence microscopy, molecular docking, and gene expression profiling. To confirm the involvement of p53, we generated CRISPR-Cas9-mediated *TP53*-knockout MCF-7 cells. Our results show that Desloratadine significantly reduced viability and induced apoptosis in wild-type MCF-7 cells, whereas these effects were greatly diminished in *TP53*-knockout MCF-7 cells. Together, these findings indicate that Desloratadine induces cell death partially through a p53-dependent apoptotic pathway, supporting its potential as a repurposed drug candidate for breast cancer therapy.

## 2. Materials and Methods

### 2.1. Chemicals

MTT, SYBR Green RT master mix, cDNA synthesis kit, and primers were purchased from Beijing TsingKe Biological Technology, Beijing, China. DMEM medium, Trypsin-EDTA, and FBS were obtained from Gibco (Waltham, MA, USA). Streptomycin and neomycin were from Amresco (Solon, OH, USA). FITC-annexin V/PI was purchased from Invitrogen (Waltham, MA, USA). Propidium iodide (PI) and caspase inhibitors were obtained from Sigma (St. Louis, MO, USA). Desloratadine was collected from a local pharmaceutical company. HEPES and RNase A were purchased from Carl Roth, Karlsruhe, Germany.

### 2.2. Cell Culture and Treatment Protocol

Two established cell lines were utilized in this study: MCF-7 and non-cancerous Human Embryonic Kidney (HEK) 293T, all procured from ATCC. Cells were cultured in DMEM medium with high glucose, L-glutamine, pyridoxine-HCl, and sodium pyruvate. Sodium bicarbonate and HEPES, 1% PSN (penicillin–streptomycin–neomycin), and 10% FBS were added subsequently. Cells were cultured at 37 °C with 5% CO_2_ and routinely passaged at 80% confluency to ensure optimal growth. For MTT assays, cells were seeded in 96-well plates at optimized densities: MCF-7 and HEK293T at 1.0 × 10^4^ cells/well. 24 h later, MCF-7 cells were treated with varying concentrations of Desloratadine (4–64 μg/mL) and standard drug 5-Florouracil (8–64 μg/mL), and HEK293T cells were treated with 16–128 μg/mL Desloratadine for 48 h. After treatment, DMEM medium was carefully aspirated and replaced with MTT solution for viability assessment. Next 2 h of incubation, visible crystals formed, which were dissolved in the mixture of isopropanol and HCl following the removal of aliquots from each well. After 30 min, we recorded the absorbance of the magenta dye solution at 570 nm using a microplate reader (BioTek 800TS, BioTek Instruments, Inc., Winooski, VT, USA). Then, the IC_50_ values were calculated.

### 2.3. Determination of IC_50_ Value

The IC_50_ value was calculated using the linear interpolation method [25]. The percentage of inhibition was first determined at various concentrations of the test compound (64, 32, 16, 8, and 4 μg/mL). The two concentrations between which 50% inhibition occurred were identified. The IC_50_ value was then calculated using the following formula:

IC_50_ = *C*_1_ + (*C*_2_ − *C*_1_)/(*I*_2_ − *I*_1_) × (50 − *I*_1_) (1)

where the following applies:

*C*_1_ and *C*_2_ are two concentrations between which 50% inhibition lies.

*I*_1_ and *I*_2_ are the corresponding inhibition percentages at *C*_1_ and *C*_2_.

### 2.4. Morphological Study Using a Fluorescence Microscope

To assess morphological changes in MCF-7 cells following treatment with Desloratadine, 4 × 10^4^ cells were seeded into each well of a 24-well culture plate and treated with 16 μg/mL. Desloratadine for 48 h. After removing the culture medium, the cells were rinsed with PBS and incubated with Hoechst 33342 for approximately 20 min in the dark. Cellular morphology was examined using a fluorescence microscope (Olympus IX71, Olympus Corporation, Seoul, Korea), and images were captured and saved in ‘.jpg’ format.

### 2.5. Observation of Reactive Oxygen Species (ROS) Changes

To detect changes in ROS levels, MCF-7 cells were treated with 16 μg/mL Desloratadine and stained with 2′,7′-dichlorofluorescein diacetate (DCFH-DA). Cells were cultured in a 24-well plate and treated with Desloratadine for 48 h, as previously described. Following treatment, the cells were washed with serum-free medium and incubated with diluted DCFH-DA (1:1000) at 37 °C for 20 min. ROS levels were then assessed using a fluorescence microscope.

### 2.6. Detection of Early and Late Apoptosis by Fluorescence Microscopy and Flow Cytometry

MCF-7 cells were seeded into 6-well and 24-well culture plates for flow cytometry and fluorescence microscopy, respectively, and treated with 16 μg/mL Desloratadine as described above. After 24 h, the cells were detached from the 6-well culture plate and washed twice with PBS and annexin V binding buffer. Similarly, after 48 h of Desloratadine treatment, the cells in the 24-well culture plate were washed without detaching.Subsequently, the cells were stained with FITC-annexin V and propidium iodide (PI) for 20 min in the dark at room temperature. Early and late apoptotic cells were then detected using an Olympus IX71 fluorescence microscope and a CytoFLEX flow cytometer (Beckman Coulter, Inc., Brea, CA, USA).

### 2.7. Cell Cycle Analysis by Flow Cytometry

MCF-7 cells were cultured in T-25 flasks and treated with 16 µg/mL of Desloratadine for 24 h and 48 h. Then, the cells were detached with Trypsin-EDTA, rinsed with PBS, and resuspended in 1 mL of ice-cold PBS. Ice-cold 70% ethanol (3mL) was added dropwise with continuous shaking to fix the cells, which were subsequently stored at −20 °C. Before experiments, ethanol was removed by washing with PBS, and the cells were incubated with RNase A at 37 °C for 30 min. Then PI was added, and the cell cycle distribution was assessed using a CytoFLEX flow cytometer.

### 2.8. Gene Expression by Real-Time PCR

MCF-7 cells were exposed to a 16 μg/mL concentration of Desloratadine for 48 h. Total RNA was extracted using reagents obtained from FAVORGEN (Pingtung, Taiwan, China) according to the supplied protocol. Then, cDNA was synthesized using a cDNA preparation kit (TsingKe, Beijing, China). After that, cDNA templates were mixed with 2× SYBR Green master mix (TsingKe, China) following the manufacturer’s specifications. The PCR conditions were set to 95 °C for 3 min followed by 40 cycles at 95 °C for 10 s and 60 °C for 30 s as supplied. Expression levels of *TP53*, *FAS*, *BAX*, *MAPK*, *EGFR*, *BCL2*, *PARP1*, *STAT3*, and *NF-κB* were detected using a CFX96 Real-Time PCR detection system. 18s rRNA was used as the primary housekeeping gene. The final Real-Time PCR data were analyzed using the double delta CT method. Primer’s list is shown in Table 1.

### 2.9. Involvement of Caspases in the Apoptosis Process

The effects of caspases on the cytotoxicity of Desloratadine against MCF-7 cells were evaluated using caspase-7 inhibitor (z-DEVD-fmk), caspase-8 inhibitor (z-IETD-fmk), and caspase-9 inhibitor (z-LEHD-fmk). Briefly, 1.0 × 10^4^ MCF-7 cells were seeded in each well of a 96-well culture plate. After 48 h, the cells were incubated with 2 μM of each caspase inhibitor for 90 min at 37 °C with 5% CO_2_. Subsequently, the cells were treated with 32.0 μg/mL of Desloratadine for 48 h. Cytotoxicity was assessed using the MTT assay. Cells without Desloratadine or caspase inhibitors served as the control.

### 2.10. In Silico Study

The interactions of Desloratadine with the p53, FAS, and NF-κB proteins were evaluated through molecular docking simulations. The 3D structures of the p53, FAS, and NF-κB proteins were obtained from the Protein Data Bank (https://www.rcsb.org/, accessed on 1 November 2025) using PDB codes 2OCJ, 1DDF, and 1SVC, respectively. To reduce undesired interactions with the receptors, all water molecules and heteroatoms were removed from the protein structures using Biovia Discovery Studio 2021. The proteins were then prepared by converting the PDB files into pdbqt format for docking. Desloratadine (CID no. 124087) was retrieved in 3D SDF format from the PubChem database (https://pubchem.ncbi.nlm.nih.gov/, accessed on 1 November 2025), minimized, and subsequently converted into pdbqt format using PyRx tools.

Docking simulations were carried out using PyRx Autodock Vina to calculate the binding affinities between Desloratadine and the target proteins. The binding interactions, including hydrogen bonds, Pi-Alkyl, Pi-Sigma interactions, and hydrophobic contacts, were thoroughly analyzed using BIOVIA Discovery Studio 2021 (version 21.1.0.20298). The binding energy values were calculated, and specific interactions were identified for each protein–ligand complex.

### 2.11. CRISPR-Cas9 Knockout of TP53

A lenti-multi-CRISPR plasmid vector containing three sgRNAs targeting *TP53* and a puromycin resistance gene was obtained from Addgene (Plasmid #85402, https://www.addgene.org/85402/, accessed on 1 November 2025). The selected sgRNAs for *TP53* knockout were as follows:

gRNA 1: TCTCGAAGCGCTCACGCCCA (antisense strand)

gRNA 2: GCAGTCACAGCACATGACGG (sense strand)

gRNA 3: TCCTCAGCATCTTATCCGAG (sense strand)

The Lenti-multi-CRISPR vector was propagated in calcium-competent *Escherichia coli* DH5α. After puromycin selection, plasmid DNA was extracted using the HiElute Miniprep Spin Column Kit (HiMEDIA), and agarose gel electrophoresis was performed to verify the plasmid quality.

MCF-7 cells (1 × 10^4^ cells/well) were seeded in a 96-well culture plate. After 24 h, the cells were transfected with the CRISPR construct using the CANFAST transfection reagent (CANFAST, Sant Cugat del Vallès, Spain), following the manufacturer’s protocol. After an additional 48 h, cells were subjected to puromycin (1 μg/mL) selection for 24 h, Surviving cells were transferred to fresh wells on the same plate a few days later, and puromycin selection was repeated subsequently The cells were then progressively scaled up to 24-well and 6-well plates, and ultimately to a T25 culture flask. Transfection efficiency was assessed using a GFP-expressing control vector provided by CANFAST. Wild-type MCF-7 cells were treated with puromycin (1 μg/mL) for 24 h to assess its effect on *TP53* expression.

To confirm *TP53* knockout, total RNA was extracted from *TP53*-knockout, wild-type, and puromycin-treated MCF-7 cells. cDNA was synthesized, and qPCR was performed as described previously using 18s rRNA as housekeeping gene (primer in Table 1). In addition to the *TP53* primers shown in Table 1, the following *TP53* primer pair was also used:

Forward: 5′-TATCCAGTCGTCTACTATGCCTC-3′

Reverse: 5′-CATTTGAAGGACTCCAATCAGGG-3′

Following *TP53* knockout, Desloratadine was applied to the *TP53*-knockout MCF-7 cells (1 × 10^4^ cells/well) at concentrations ranging from 4 to 64 µg/mL for 48 h, and cytotoxicity was assessed using an MTT assay as described earlier.

### 2.12. Statistical Analysis

Quantitative data are presented as means ± standard deviations (SD). Data was organized and subjected to basic calculations using Microsoft Excel, while detailed graphs and curve fitting were performed using Microsoft Excel and GraphPad Prism (v8.0.2). Intergroup variability was assessed using a one-way ANOVA with Tukey’s post hoc test in SPSS Statistics (v21.0). Results with *p*-values below 0.05 were considered statistically significant.

## 3. Results

### 3.1. Desloratadine Inhibits MCF-7 Cell Growth and Induces Apoptotic Morphology

**Figure 1:**
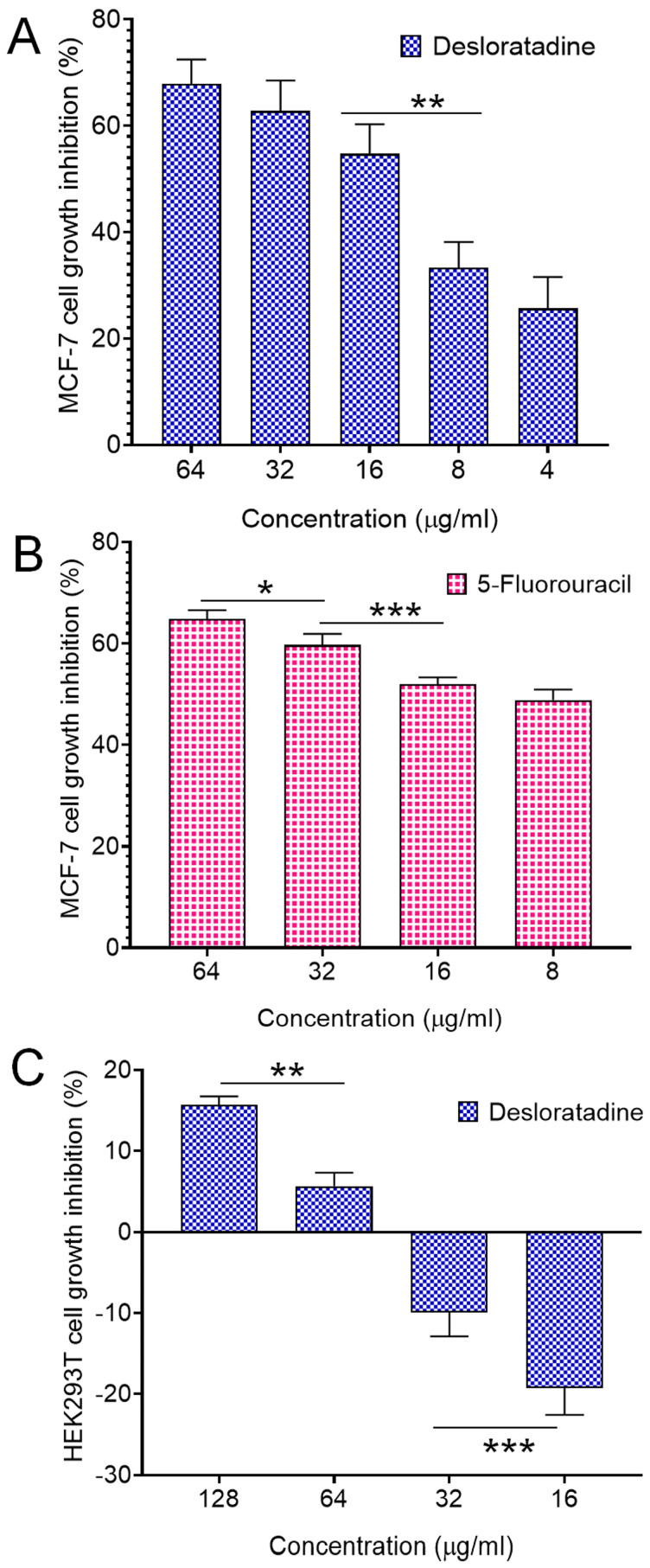
Cell growth inhibition by Desloratadine and 5-Fluorouracil. MCF-7 cells were treated with various concentrations of Desloratadine and 5-Fluorouracil for 48 h. HEK293T cells were also treated with different concentrations of Desloratadine. Cell growth inhibition was determined using the MTT assay (performed in triplicate assays), and the IC_50_ values were calculated from the results. (**A**,**B**) represent the cytotoxic effects of Desloratadine and 5-Fluorouracil on MCF-7 cells, respectively, while (**C**) represents the cytotoxic effect of Desloratadine on HEK293T cells. Three individual experiments were conducted in (**A**), while (**B**,**C**) were single experiments. Each experiment was performed in triplicate. Data represents the standard deviation (SD).* *p* < 0.05, ** *p* < 0.01, *** *p* < 0.001.

**Figure 2:**
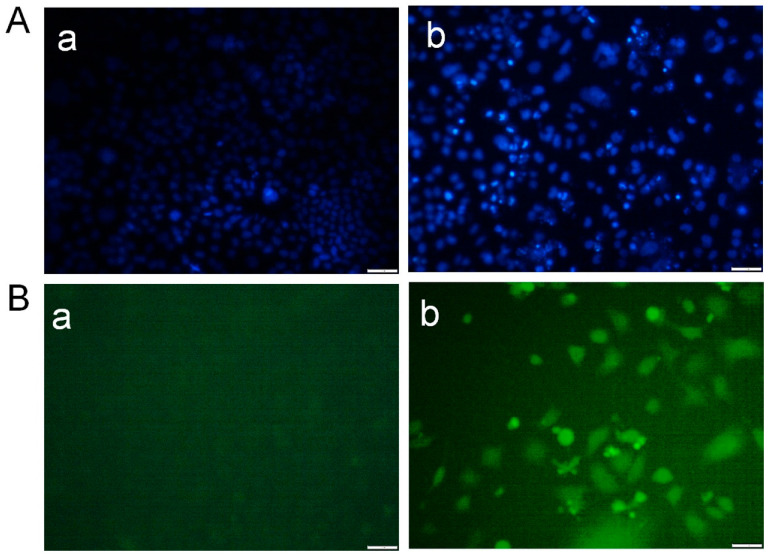
Detection of morphological alterations in MCF-7 cells after treatment with Desloratadine. MCF-7 cells were treated with 16 µg/mL Desloratadine for 48 h. After treatment, the cells were washed with PBS and stained with Hoechst 33342 dye for 20 min. In a separate experiment, the cells were washed with serum-free medium and incubated with DCFH-DA dye for 20 min in the dark. Finally, images were captured for both experiments. (**A**) Morphological changes observed after staining with Hoechst 33342 dye. (**B**) Images obtained after staining with DCFH-DA dye. In both (**A**,**B**): (a) fluorescence image of control MCF-7 cells, and (b) fluorescence image of Desloratadine-treated MCF-7 cells. All images were captured at 20× magnification. The white scale bar represents 50 µm. (n = 3).

Treatment of MCF-7 breast cancer cells with Desloratadine resulted in a dose-dependent inhibition of growth. At 64 μg/mL for 48 h, cell growth was inhibited by 67.8%, while inhibition decreased to 25.7% at 4 μg/mL (Figure 1A). 5-fluorouracil (5-FU) was used as a standard drug that inhibited growth by 64.84% at 64 μg/mL and 48.86% at 16 μg/mL (Figure 1B). In contrast, non-cancerous HEK293T cells exhibited minimal sensitivity to Desloratadine, with only 15.6% inhibition observed at 128 μg/mL and 5.6% at 64 μg/mL. Notably, lower concentrations of 32 μg/mL and 16 μg/mL increased viability by 9.9% and 19.2%, respectively (Figure 1C). The IC_50_ values were calculated to be 10.88 μg/mL for 5-FU and 14.2 μg/mL for Desloratadine. In HEK293T cells, Desloratadine did not induce significant cytotoxicity even at the highest tested concentration (128 µg/mL), with a maximum inhibition of only 15.6%. As such, an IC_50_ value could not be determined in this cell line. This indicates a favorable selectivity profile for Desloratadine against cancer cells. Consistent with these findings, fluorescence microscopy using Hoechst 33342 staining revealed distinct apoptotic features in Desloratadine-treated MCF-7 cells, including nuclear condensation and fragmentation, which were absent in untreated controls (Figure 2A). These morphological changes represent hallmarks of apoptosis and support the conclusion that Desloratadine exerts its cytotoxic effect through induction of apoptotic pathways in breast cancer cells.

### 3.2. Desloratadine Induces ROS Generation and Detection of Early and Late Apoptosis

**Figure 3:**
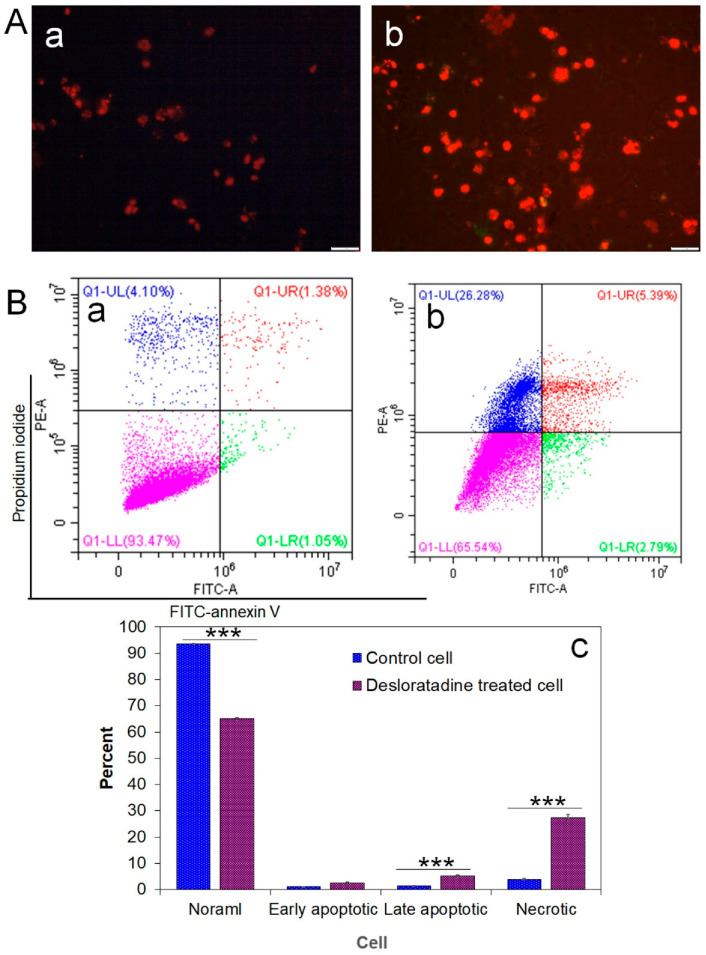
Detection of early and late apoptosis in MCF-7 cells after treatment with Desloratadine. MCF-7 cells were treated with 16 µg/mL Desloratadine for 48 h for fluorescence microscopy and for 24 h for flow cytometric analysis. (**A**) Morphological changes observed after annexin V/PI staining. (a) fluorescence image of control MCF-7 cells; (b) fluorescence image of Desloratadine-treated MCF-7 cells. (**B**) Detection of apoptosis by flow cytometry. (a,b) represent untreated and Desloratadine-treated cells, respectively. Magenta, green, red, and blue dots indicate normal cells, early apoptotic cells, late apoptotic cells, and necrotic cells/debris, respectively. (**C**) Data were presented as mean ± SD from three technical triplicates (*** *p* < 0.001).

Intracellular ROS generation was assessed using the fluorescent probe DCFH-DA. Desloratadine-treated MCF-7 cells exhibited a marked increase in fluorescence intensity, indicating elevated ROS levels, whereas untreated cells showed no significant ROS production (Figure 2B). To further examine apoptosis, FITC-annexin V/PI staining was performed. Untreated MCF-7 cells displayed only minimal apoptotic populations, while Desloratadine treatment for 48 h resulted in a substantial increase in both early and late apoptotic cells as observed by fluorescence microscopy (Figure 3A). Consistent with these findings, flow cytometry analysis after 24 h of treatment demonstrated a significant rise in late apoptotic populations, accompanied by an increase in necrotic cells (Figure 3B,C).

### 3.3. Effect of Caspase Inhibitors in the Apoptosis Process and Modulation of Apoptotic and Survival Gene Expression in MCF-7 Cells

**Figure 4:**
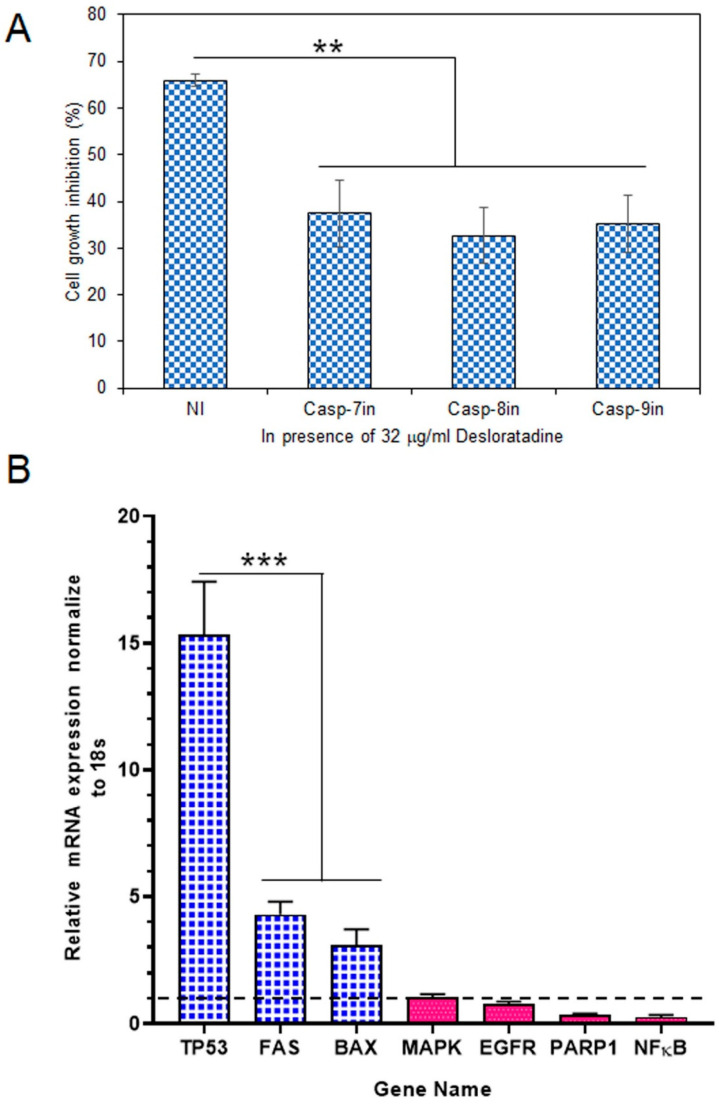
Effects of caspase inhibitors in Desloratadine-treated cells and the effects of Desloratadine on gene expression and cell cycle phases. (**A**) MCF-7 cells were treated with 32 µg/mL Desloratadine in the presence or absence of caspase inhibitors for 48 h, followed by MTT assay. ‘NI’ indicates cells treated with 32 µg/mL Desloratadine alone. Casp-7in, Casp-8in, and Casp-9in represent treatment with Desloratadine in the presence of caspase-7, caspase-8, and caspase-9 inhibitors, respectively. Data are expressed as mean ± SD of four replicate wells. Statistical significance was determined in comparison with the ‘NI’ group. (**B**) Relative mRNA expression of *TP53*, *FAS*, *BAX*, *MAPK*, *EGFR*, *PARP1*, and *NF-κB* was measured by qPCR. Each gene was analyzed in at least three replicate wells, and values were averaged. Data are presented as mean ± SD. The dotted line indicates baseline expression (1.0). Statistical significance was determined in comparison with the control group (** *p* < 0.01, *** *p* < 0.001).

To determine whether caspases mediate this cytotoxic effect, MCF-7 cells were treated with Desloratadine in the presence of specific caspase inhibitors. At 32 μg/mL, Desloratadine inhibited proliferation by approximately 65.93.0%, but this inhibition was reduced by 37.4% with a caspase-7 inhibitor, 32.64% with a caspase-8 inhibitor, and 35.27% with a caspase-9 inhibitor (Figure 4A). These results demonstrate that Desloratadine promotes cell death in MCF-7 cells through caspase-dependent apoptotic pathways involving both intrinsic and extrinsic mechanisms.

Gene expression analysis revealed that Desloratadine treatment altered the balance between pro-apoptotic and survival-related genes in MCF-7 cells. Consistent with the observed ROS generation, caspase activation, and apoptotic morphology, Desloratadine strongly induced a ~15-fold increase in *TP53* expression, highlighting activation of the p53 pathway. Other pro-apoptotic genes, including *FAS* and *BAX*, were also significantly upregulated, supporting the involvement of both intrinsic and extrinsic apoptotic mechanisms. In contrast, several oncogenic and survival-related genes—such as *EGFR*, *PARP1*, and *NF-κB*—were markedly downregulated, and no change in *MAPK* expression was observed (Figure 4B). Moreover, *STAT3* and *BCL2* expressions were not detected following Desloratadine treatment, further underscoring the suppression of survival pathways. Together, these transcriptional changes align with the cellular phenotypes and confirm that Desloratadine promotes apoptosis by reinforcing pro-apoptotic signaling while simultaneously inhibiting oncogenic and survival drivers.

### 3.4. Desloratadine Induces Cell Cycle Arrest and Shows Strong Binding Affinity for Apoptotic Regulators

**Figure 5:**
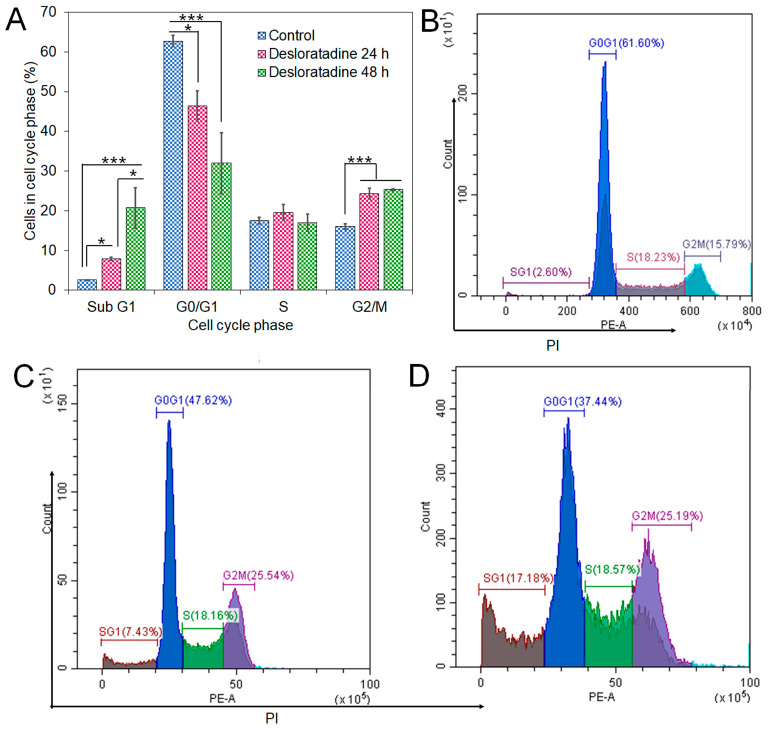
Effects of Desloratadine on cell cycle distribution in MCF-7 cells. The percentages of cells in each phase of the cell cycle were evaluated by flow cytometry, as shown in panel (**A**). Panels (**B**–**D**) show representative flow cytometry histograms for the control and Desloratadine-treated cells after 24 h and 48 h, respectively. The *X*-axis represents the intensity of PI staining, which is proportional to cellular DNA content, and the *Y*-axis represents the number of cells. Data are based on mean values from three independent experiments and are presented as mean ± SD. (* *p* < 0.05, *** *p* < 0.001).

**Figure 6:**
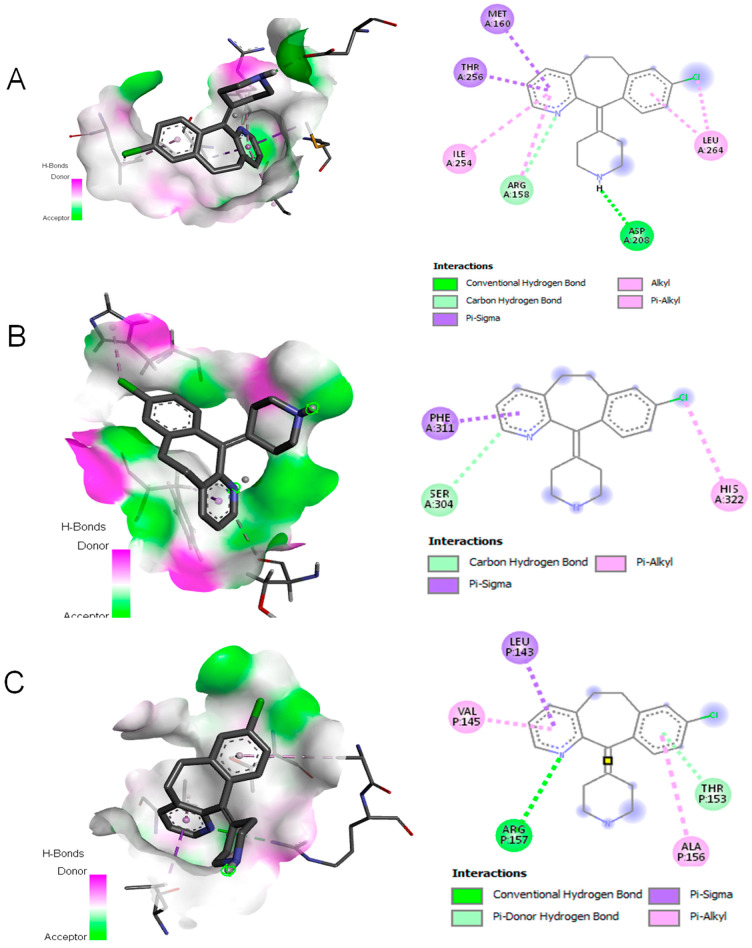
Molecular docking simulation. (**A**) Interaction of Desloratadine with p53; (**B**) interaction with FAS; (**C**) interaction with a single chain of NF-κB.

Flow cytometry analysis revealed that Desloratadine treatment altered the distribution of MCF-7 cells across the cell cycle. In untreated cells, the sub-G_1_, G_0_/G_1_, S, and G_2_/M phases were 2.69%, 62.19%, 17.68%, and 15.5%, respectively. After treatment with Desloratadine for 24 h, the sub-G_1_ population increased markedly to 7.88%, accompanied by an elevation of the G_2_/M population to 24.26%, while the G_0_/G_1_ population decreased to 46.36%. When the treatment time increased to 48 h, the sub-G_1_ phase increased remarkably to 20.74%, with the G_0_/G_1_ phase decreasing to 32.01%. The G_2_/M phases remained nearly unchanged compared to 24 h treatment (Figure 5A,B). These results indicate that Desloratadine induces apoptosis and causes G_2_/M phase arrest in MCF-7 cells in a time-dependent manner. To further explore the molecular basis of these effects, in silico docking studies were performed. Desloratadine exhibited the highest binding affinity for p53, with a binding energy of −7.0 kcal/mol, forming one hydrogen bond with ARG158 and ASP208 and sigma and alkyl interactions with four amino acids (Figure 6A). A slightly lower binding energy of −6.8 kcal/mol was observed for FAS, where one hydrogen bond was formed with SER304, along with Pi-Sigma and Pi-Alkyl interactions with PHE311 and HIS322, respectively (Figure 6B). Binding to NF-κB was lower (−6.5 kcal/mol), involving one hydrogen bond with ARG157 and additional stabilizing interactions (Figure 6C). These docking results support the experimental findings by suggesting that Desloratadine directly interacts with apoptotic regulators to promote cell cycle arrest and apoptosis.

### 3.5. CRISPR-Cas9 Disruption of TP53 Confirms Its Role in Desloratadine-Mediated Apoptosis

**Figure 7:**
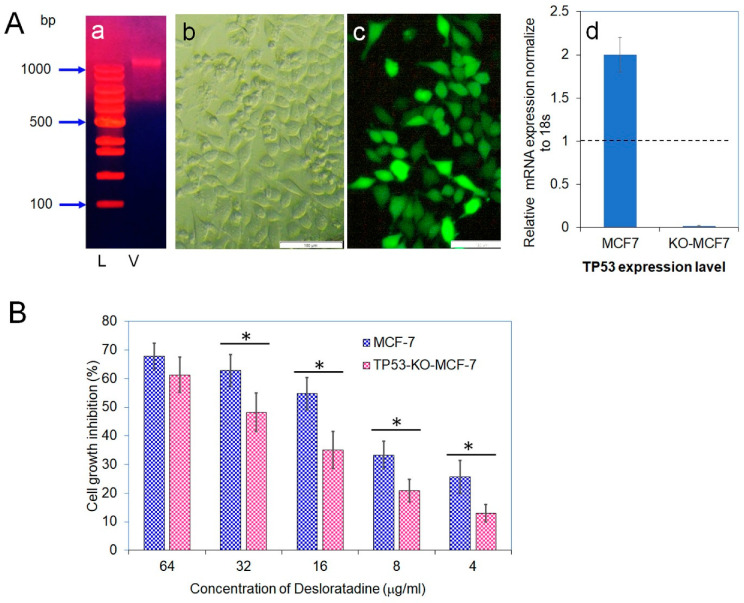
Vector isolation, transfection efficiency, *TP53* gene expression, and cytotoxicity of Desloratadine. (**A**) (a) Vector isolated from *Escherichia coli* DH5α. Lane 1: 100 bp DNA ladder (L); Lane 2: isolated vector (V). (b,c) Light and fluorescence microscopic images, respectively, showing expression of green fluorescent protein (GFP) in *TP53*-KO-MCF-7 cells 48 h after transfection with the GFP control vector. Magnification: 10×; white bar = 100 µm. (d) Relative mRNA expression of *TP53* was measured by qPCR. Each gene was analyzed in at least three replicate wells, and values were averaged. Data are presented as mean ± SD. The dotted line indicates baseline expression (1.0). Statistical significance was determined in comparison with the control. (**B**) Cytotoxicity of Desloratadine at different concentrations in *TP53*-KO-MCF-7 cells and wild-type MCF-7 cells (data for wild-type cells were previously presented in Figure 1A). Data are expressed as mean ± SD of replicate wells. Statistical comparisons were made between *TP53*-KO and wild-type cells at each concentration. * *p* < 0.05.

Because Desloratadine-induced cytotoxicity appeared to involve *TP53*, we disrupted the *TP53* gene in MCF-7 cells using a Lenti-multi-CRISPR vector carrying three sgRNAs against *TP53* along with a puromycin resistance marker (Figure 7A). Stable incorporation was confirmed by puromycin selection, and delivery efficiency was validated with a GFP-expressing control vector. Quantitative analysis demonstrated that *TP53* transcript levels in wild-type MCF-7 cells were set as baseline (1.0), whereas expression in *TP53*-knockout cells was nearly abolished (0.019), confirming efficient gene disruption. Furthermore, treatment of wild-type MCF-7 with puromycin caused a twofold increase in TP53 expression. To evaluate the functional impact of *TP53* loss, both wild-type and *TP53*-knockout MCF-7 cells were exposed to Desloratadine. Wild-type cells displayed dose-dependent growth inhibition with an IC_50_ of 14.2 μg/mL, whereas *TP53*-knockout cells exhibited markedly reduced sensitivity with an IC_50_ of 36.4 μg/mL. At 64 μg/mL, Desloratadine inhibited 61.3% of *TP53*-knockout cell growth compared with substantially higher inhibition in wild-type cells, and at 4 μg/mL, inhibition was only 13.0% (Figure 7B). These findings confirm both the successful knockout of the *TP53* gene and its critical role in mediating the cytotoxic effects of Desloratadine in MCF-7 cells.

## 4. Discussion

Drug repurposing is increasingly recognized as a powerful approach to accelerate the development of novel therapeutics by uncovering new uses for existing drugs with well-established safety profiles [26,27]. Within oncology, this strategy provides a cost-effective route to identify agents with anticancer properties that might otherwise remain overlooked. Our findings highlight Desloratadine, a widely prescribed second-generation antihistamine, as a candidate with promising anticancer potential against breast cancer cells. Although less potent than the chemotherapeutic drug 5-fluorouracil, Desloratadine demonstrated selective cytotoxicity in MCF-7 cells while showing minimal toxicity toward non-cancerous HEK293T cells, an encouraging feature for drug repositioning.

The selective cytotoxic activity of Desloratadine suggests an ability to discriminate between malignant and non-malignant cells, a property highly desirable in anticancer therapy. At clinically relevant concentrations, Desloratadine impaired cancer cell viability while largely sparing normal cells. This observation parallels reports of other repurposed drugs that exert context-dependent cytotoxicity in cancer cells while maintaining acceptable safety profiles [28]. Such selectivity could reduce adverse effects associated with conventional chemotherapeutics, supporting further investigation of Desloratadine in preclinical cancer models.

Mechanistic insights from this study demonstrate that Desloratadine induces apoptosis, a programmed cell death essential for eliminating damaged cells [29]. The observed apoptotic features, including nuclear condensation, DNA fragmentation, and accumulation of sub-G_1_ cells, are consistent with apoptosis induced by established anticancer drugs [30]. Apoptosis was attenuated by caspase inhibition, implicating caspase-7, -8, and -9 in the process. This suggests the engagement of both intrinsic mitochondrial and extrinsic death receptor pathways [31,32]. The involvement of ROS further reinforces the interpretation that Desloratadine disrupts redox homeostasis, leading to oxidative stress–mediated apoptosis, a mechanism increasingly exploited in cancer therapy [33,34].

Gene expression analyses provided additional mechanistic support. Upregulation of *TP53*, *BAX*, and *FAS* suggests coordinated activation of apoptosis [35,36]. p53 orchestrates cell cycle arrest and apoptosis in response to stress. Its upregulation aligns with increased expression of downstream effectors such as *BAX* and *FAS*, indicating that Desloratadine-induced apoptosis may be partly mediated through p53-dependent signaling. Conversely, there is no change in *MAPK* expression and downregulation of *EGFR*, *PARP1*, and *NF-κB*, along with complete loss of *STAT3* and *BCL2*, which highlights suppression of pro-survival pathways. NF-κB and STAT3 promote proliferation and resistance to apoptosis [37,38]. Their suppression likely sensitizes cells to apoptotic signals, while the absence of BCL2 further favors the balance toward cell death. Collectively, these transcriptional shifts illustrate how Desloratadine enhances pro-apoptotic signaling while disrupting survival pathways, producing a dual assault on breast cancer cells. Desloratadine demonstrated a dual cytotoxic effect by inducing both apoptosis and mTOR/AMPK-dependent autophagy in U251 human glioblastoma cells as well as in primary glioblastoma cell culture [39].

Molecular docking provided complementary evidence, showing strong binding affinity of Desloratadine for p53, FAS, and NF-κB, suggesting activation of pro-apoptotic regulators and disruption of survival proteins. While in silico predictions cannot confirm direct binding, their consistency with transcriptional and phenotypic data supports these pathways as relevant targets. The functional role of p53 was further validated by CRISPR-Cas9 knockout, where *TP53*-deficient cells showed markedly reduced sensitivity to Desloratadine, with the IC_50_ increasing nearly threefold compared to wild-type cells. This establishes p53 as a key mediator of Desloratadine-induced apoptosis and aligns with previous evidence that p53 loss confers resistance to therapy [40]. These findings suggest that Desloratadine may be particularly effective in cancers retaining functional *TP53*, such as luminal breast cancers.

We also acknowledge the limitations of this study. These findings are based on a single breast cancer cell line, and validation in additional models, including triple-negative and HER2-positive breast cancers, is needed. In vivo studies will be essential to determine whether Desloratadine achieves effective plasma concentrations without toxicity. Pharmacokinetic and pharmacodynamic profiling will be critical for assessing clinical feasibility. Moreover, while docking suggests potential binding to p53, FAS, and NF-κB, experimental validation using biophysical assays will be required.

In summary, our study demonstrates that Desloratadine exerts selective anticancer effects in breast cancer cells by inducing p53-dependent apoptosis. The drug activates both intrinsic and extrinsic apoptotic pathways, disrupts survival signaling, and shows strong selectivity for malignant cells over non-malignant cells. The dependency on *TP53* underscores its potential utility in cancers retaining functional *TP53*, while minimal toxicity to non-cancerous cells suggests a favorable safety profile. Collectively, these findings identify Desloratadine as a promising repurposed drug candidate for breast cancer therapy. Further in vivo validation and translational studies are warranted to explore its potential integration into existing treatment regimens.

## Figures and Tables

**Table 1:** Primer List.

18s rRNA	F	GTAACCCGTTGAACCCCATT
	R	CCATCCAATCGGTAGTAGCG
BAX	F	CATATAACCCCGTCAACGCAG
	R	GCAGCCGCCACAAACATAC
PARP-1	F	GGCCTCGGTGGATGGAATG
	R	GCAAACTAACCCGGATAGTCTCT
EGFR	F	AGGCACGAGTAACAAGCTCAC
	R	ATGAGGACATAACCAGCCACC
MAPK4	F	CGGTGTCAATGGTTTGGTGC
	R	GACGATGTTGTCGTGGTCCA
*TP53*	F	GCCCAACAACACCAGCTCCT
	R	CCTGGGCATCCTTGAGTTCC
STAT3	F	CAGCAGCTTGACACACGGTA
	R	AAACACCAAAGTGGCATGTGA
FAS	F	AGCTTGGTCTAGAGTGAAAA
	R	GAGGCAGAATCATGAGATAT
BCL2	F	AGTTATCGGCTTCAGTGGTCT
	R	CTGCCCGCTTCCTAGCTTG
NF-κB	F	GAATTGCTGCTTCGGAATGGA
	R	CATGCGGGCATCTACCTGG

## Data Availability

The original contributions presented in this study are included in the article. Further inquiries can be directed to the corresponding author.

## Abbreviations

MTT	(3-[4:5-dimethylthiazole-2-yl]-2,5-diphenyltetrazolium bromide)
DMEM	Dulbecco’s Modified Eagle Medium
HEPES	(4-(2-hydroxyethyl)-1-piperazineethanesulfonic acid)
FITC	Fluorescein isothiocyanate
IC_50_	Half maximal inhibitory concentration
PBS	Phosphate-buffered saline
*PARP1*	Poly(ADP-ribose) polymerase 1
*EGFR*	Epidermal growth factor receptor
*MAPK*	Mitogen-Activated Protein Kinase
*STAT3*	Signal Transducer and Activator of Transcription 3
*ERK*	Extracellular Signal-Regulated Kinase
ANOVA	One-way analysis of variance
FBS	Fetal bovine serum
